# Supplementary material for: Pathways for balancing CO2 emissions and sinks
Source: Nat Commun. 2017 Apr 13;8:14856. doi: 10.1038/ncomms14856 (PMC5399292; doi:10.1038/ncomms14856)
Supplement: Supplementary Information — Supplementary Figures, Supplementary Tables and Supplementary References [file ncomms14856-s1.pdf]

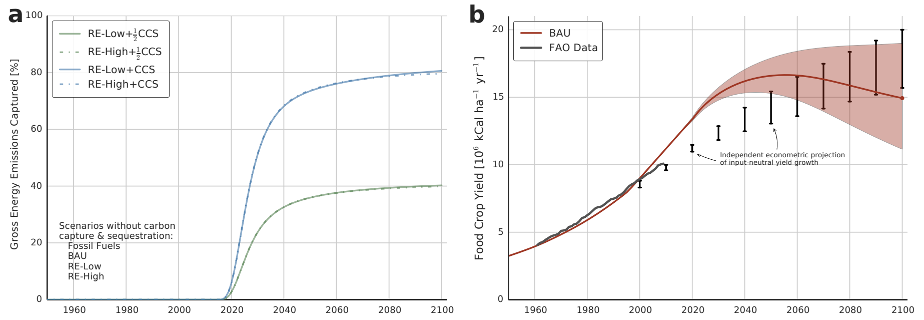

Supplementary Figure 1 CCS scale-up and agricultural yields: (**a**) Percentage of gross energy sector carbon emissions captured and permanently sequestered through CCS technology. In the six scenarios with CCS, emissions mitigation technology ramps up rapidly from 2020 through 2040. Subsequent growth in carbon capture efficiency slows due to increasing marginal costs of mitigation. (**b**) Areal *BAU* scenario crop yield projections [10^6 kCal ha^{-1} yr^{-1}]. All other scenarios show small divergence from the nominal *BAU* projection, and are contained within the boundaries of the red shaded range. Recent historical estimate, plotted in grey, is calculated from FAOSTAT data on per capita consumption, population, and agricultural area. Black bars indicate the results of an independent econometric analysis of yield growth due to input-neutral technological advancements[1].

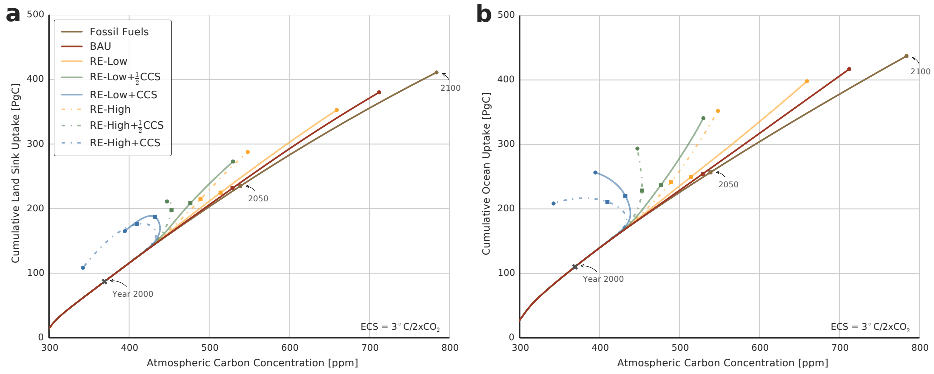

Supplementary Figure 2 Cumulative natural sink flux as a function of atmospheric carbon: cumulative land sink **(a)** and oceanic **(b)** carbon uptake [PgC] plotted as a function of atmospheric carbon concentration [ppm].

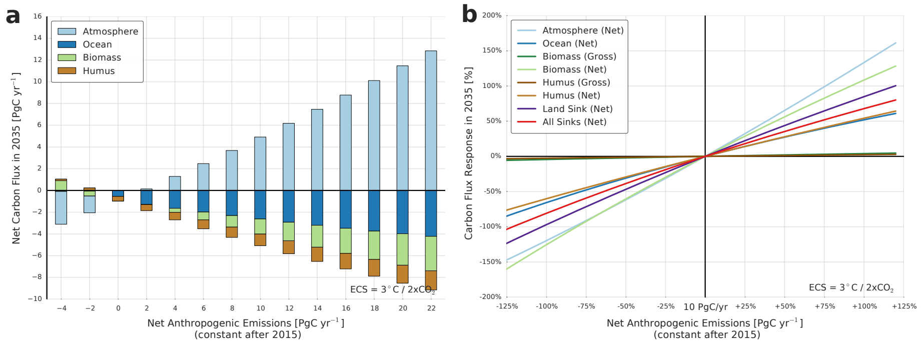

Supplementary Figure 3 Carbon cycle response to constant emissions: (a) steady-state annual net carbon uptake (flux) by atmospheric, hydrospheric, biospheric, and pedospheric sinks in year 2035 of the simulation, assuming constant net anthropogenic emissions after 2015 at the value specified on the x-axis. **(b)** Total change in net and gross carbon fluxes as function of change in annual anthropogenic emissions.

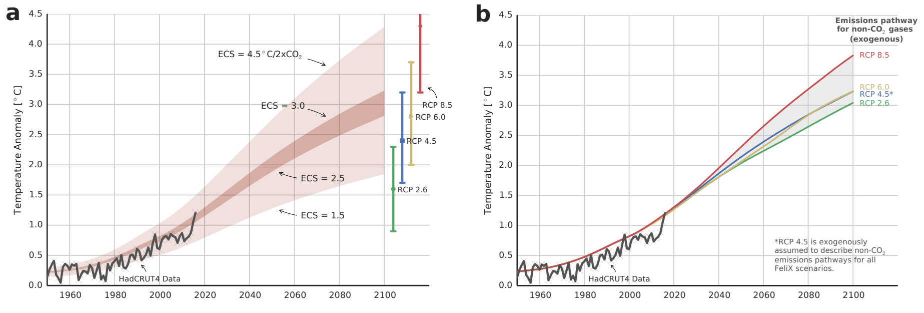

Supplementary Figure 4 Temperature anomaly sensitivity to model parameters: (a) sensitivity of ΔT to equilibrium climate sensitivity (ECS) in the *BAU* scenario. ECS quantifies the warming response to a doubling of atmospheric carbon concentration [$^\circ$C/2×CO_2]. The dark shaded region indicates 2.5 $\leq$ ECS $\leq$ 3.0, and the light shaded region indicates 1.5 $\leq$ ECS $\leq$ 4.5. Historical data from HadCRUT4 are shown in grey at left[2]. **(b)** sensitivity of ΔT to alternative non-CO_2 RCPs.

Flux		1980-89 [PgC yr^{-1}]	1990-99 [PgC yr^{-1}]	2002-11 [PgC yr^{-1}]
Fossil Fuel Emissions	Υ_{FF}	5.5 $\pm$ 0.4	6.4 $\pm$ 0.5	8.3 $\pm$ 0.7
LULUCF Emissions	Υ_{LUC}	1.4 $\pm$ 0.8	1.5 $\pm$ 0.8	0.9 $\pm$ 0.8
Renewable Energy Emissions	Υ_{RE}	–	–	–
Ocean Uptake	Ω_O	2.0 $\pm$ 0.7	2.2 $\pm$ 0.7	2.4 $\pm$ 0.7
Residual Land Sink	Ω_{LS}	1.5 $\pm$ 1.1	2.6 $\pm$ 1.2	2.5 $\pm$ 1.3
Atmospheric Increase	Ω_{Atm}	3.4 $\pm$ 0.2	3.1 $\pm$ 0.2	4.3 $\pm$ 0.2

Supplementary Table 1 Error analysis on present value of R_{AF}. All values and errors taken from IPCC WG3[3].

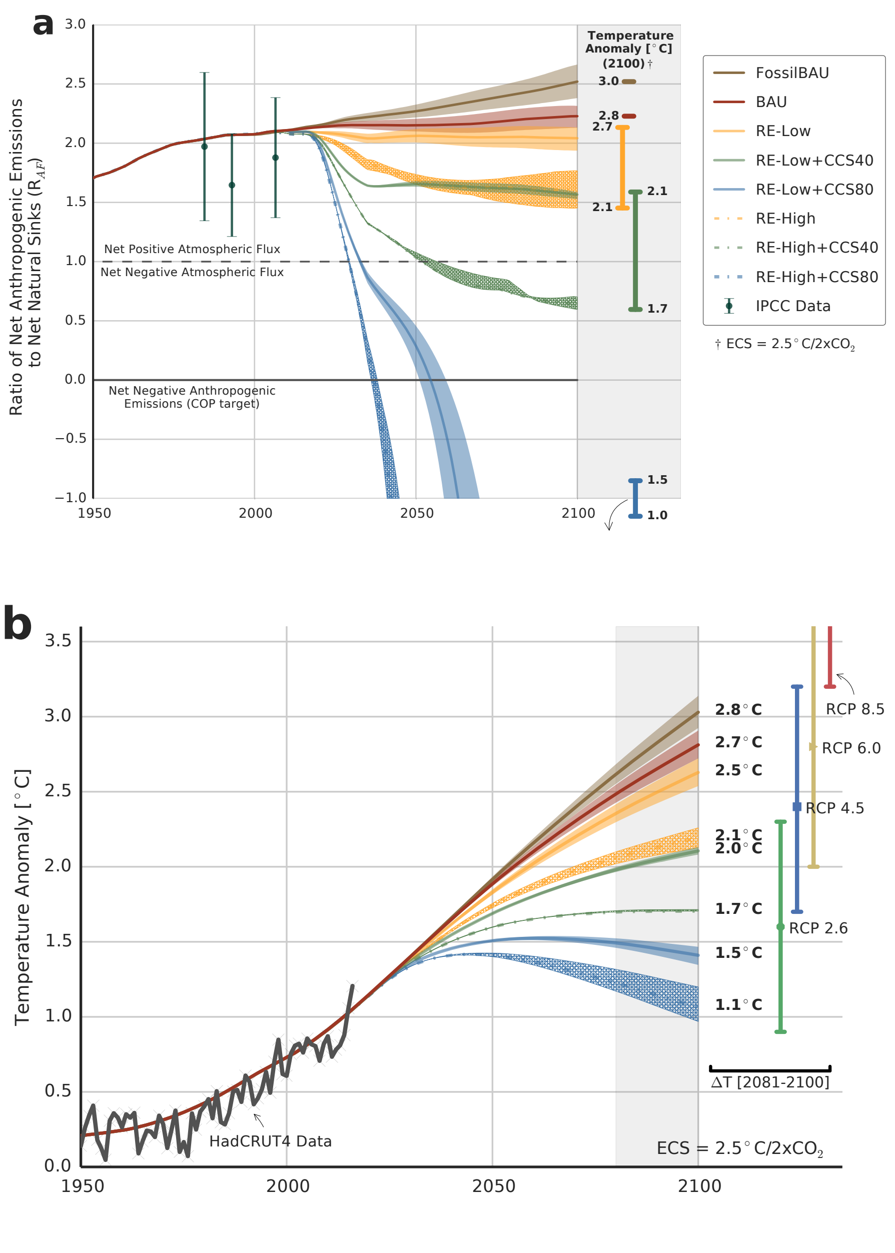

Supplementary Figure 5 Primary analysis results for ECS = 2.5°C/2×CO$_2$: (a) R_{AF}; **(b)** ΔT.

		2015	2100
	Nominal values of C_A in BAU:	*408 ppm*	*712 ppm*
**Parameter**	**Description**	**Shift [ppm]**	
World population (low)	UNDESA WPP low variant[4]	–	-77
World population (high)	UNDESA WPP high variant[4]	–	+80
Initial NPP	$NPP_I \rightarrow$ 51 PgC yr^{-1}	+15	+51
Land sink carbon residence time (high)	$\tau(t) \rightarrow \tau(t)$ + 7 years	-7	-34
Land sink carbon residence time (low)	$\tau(t) \rightarrow \tau(t)$ - 4 years	+5	+23
Energy demand (low)	+0.2% $yr^{-1}cap^{-1}$(cf. Fig. 1b)	–	-28
Energy demand (high)	-0.2% $yr^{-1}cap^{-1}$(cf. Fig. 1b)	–	+30
Equilibrium climate sensitivity	ECS $\rightarrow$ 1.5°C/2×CO_2	-5	-28
Equilibrium climate sensitivity	ECS $\rightarrow$ 2.5°C/2×CO_2	-1	-8
Equilibrium climate sensitivity	ECS $\rightarrow$ 4.5°C/2×CO_2	+3	+21
Plantation productivity (low)	10 t(dry biomass) $ha^{-1}yr^{-1}$	–	+9
Plantation productivity (high)	20 t(dry biomass) $ha^{-1}yr^{-1}$	–	-13
Forest C sequestration (low)	$C_{For.} \rightarrow$ 82.5 tC ha^{-1}	-7	-12
Forest C sequestration (high)	$C_{For.} \rightarrow$ 137.5 tC ha^{-1}	+7	+12
Non-CO_2 emissions pathway	RCP 2.6	–	-4
Non-CO_2 emissions pathway	RCP 8.5	–	+11
Bioenergy emissions (low)	$EM_{BE} \rightarrow$ 0.000 tC t(dry biomass)$^{-1}$	–	-10
Bioenergy emissions (high)	$EM_{BE} \rightarrow$ 0.098 tC t(dry biomass)$^{-1}$	–	+10
Agricultural residues (low)	Collected from 0% of arable land	–	+4
Agricultural residues (high)	Collected from 20% of arable land	–	-5
Agricultural yields (low)	-0.14% yr^{-1} (cf. Supp. Fig. 1b)	–	+3
Agricultural yields (high)	+0.14% yr^{-1} (cf. Supp. Fig. 1b)	–	-2
Food demand–animal (low)	GDP cap^{-1} effects - 10%	–	-3
Food demand–animal (high)	GDP cap^{-1} effects + 10%	–	+3
Food demand–vegetal (low)	GDP cap^{-1} effects - 10%	–	-2
Food demand–vegetal (high)	GDP cap^{-1} effects + 10%	–	+2

Supplementary Table 2 Error analysis on atmospheric carbon concentrations (C_A) [ppm] in the BAU scenario.

		Atmospheric Carbon Flux [PgC yr^{-1}]					
		**Sources**			**Sinks**		
		Fossil Fuels	LULUC	Renewables	Ocean	Land	
**Scenario**	Year (Average)	Υ_{FF}	Υ_{LUC}	Υ_{RE}	Ω_O	Ω_{LS}	R_{AF}
**IPCC Data**[5]	(2002-2011)	+8.3 ± 0.7	+0.9 ± 0.8	—	+2.4 ± 0.7	+2.5 ± 1.3	**1.9 ± 0.2**
**FeliX Model**	(2002-2011)	+9.1	+1.2	+0.0	+2.4	+2.4	**2.1 ± 0.2**
Fossil Fuels	2100	+17.8	+0.1	+0.2	+3.6	+3.3	**2.6 ± 0.7**
BAU	2100	+11.9	+0.8	+0.7	+3.2	+2.6	**2.3 ± 0.6**
RE-Low	2100	+9.5	+0.2	+0.5	+2.9	+2.2	**2.1 ± 0.5**
RE-Low+CCS40	2100	+6.1	+0.1	-1.6	+1.9	+0.9	**1.6 ± 0.6**
RE-Low+CCS80	2100	+2.2	+0.1	-4.6	+0.3	-0.9	**-4.1 ± 5.4**
RE-High	2100	+3.5	+0.3	+1.3	+2.0	+1.0	**1.7 ± 0.6**
RE-High+CCS40	2100	+2.2	+0.3	-1.9	+1.1	-0.1	**0.7 ± 0.9**
RE-High+CCS80	2100	+0.8	+0.4	-5.5	-0.5	-1.6	**-2.1 ± 1.0**

Supplementary Table 3 Magnitude of carbon sources and sinks for all scenarios in year 2100 of the FeliX model. R_{AF} and the associated errors are calculated from Eqs. 8 and 12, respectively. In extreme low emissions scenarios, errors on R_{AF} grow due to vanishing denominators.

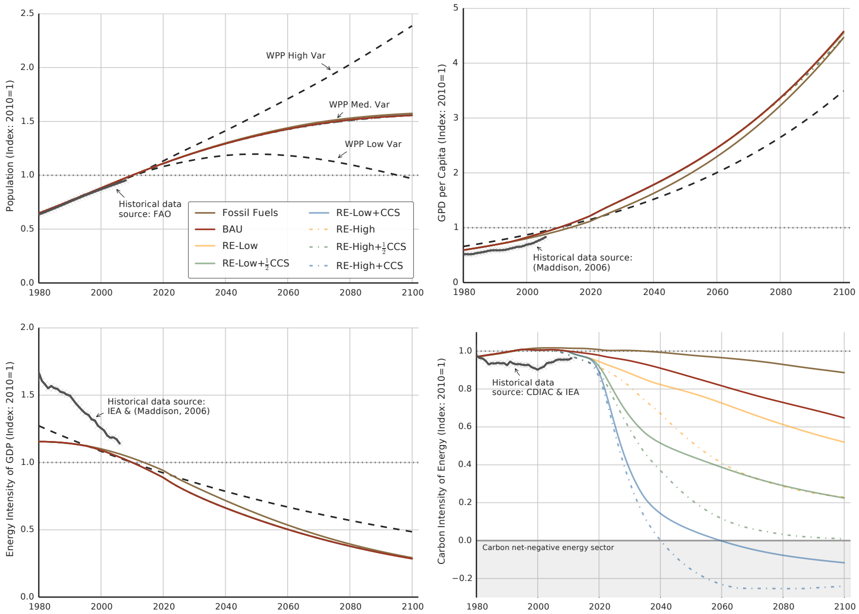

Supplementary Figure 6 Scenario results decomposed into Kaya factors: indexed to historical data from 2010. Clockwise from top left: population, show with UNDESA high, medium, and low population variants[4]; GDP per capita, shown with projection based on historical rate of increase; carbon intensity of energy; and energy intensity of GDP, shown with projection based on historical rate of decrease[6]. Where available, recent historical values are calculated from the relevant datasets and shown in grey.

Supplementary References

1. Herrero, M., Havlik, P., McIntire, J., Palazzo, A., Valin, H.: African livestock futures: Realizing the potential of livestock for food security, poverty reduction and the environment in sub-saharan africa. Technical report, Office of the Special Representative of the UN Secretary General for Food Security and Nutrition and the United Nations System Influenza Coordination (UNSIC) (2014)
2. Morice, C.P., Kennedy, J.J., Rayner, N.A., Jones, P.D.: Quantifying uncertainties in global and regional temperature change using an ensemble of observational estimates: The HadCRUT4 data set. Journal of Geophysical Research: Atmospheres **117**(D8) (2012)
3. Ciais, P., Sabine, C., Bala, G., Bopp, L., Brovkin, V., Canadell, J., Chhabra, A., DeFries, R., Galloway, J., Heimann, M., *et al.*: Carbon and other biogeochemical cycles. In: Climate Change 2013: The Physical Science Basis. Contribution of Working Group I to the Fifth Assessment Report of the Intergovernmental Panel on Climate Change, pp. 465–570. Cambridge University Press, Cambridge (2014)
4. Gerland, P., et al.: World population stabilization unlikely this century. Science **346**(6206), 234–237 (2014)
5. Moss, R.H., et al: The next generation of scenarios for climate change research and assessment. Nature **463**, 747–756 (2010)
6. Clarke, L., Jiang, K., Akimoto, K., Babiker, M., Blanford, G., Fisher-Vanden, K., Edenhofer, O., Pichs-Madruga, Y., Sokona, E., Farahani, S.: Assessing Transformation Pathways. In: Climate Change 2014: Mitigation of Climate Change. Contribution of Working Group III to the Fifth Assessment Report of the Intergovernmental Panel on Climate Change. Cambridge University Press, Cambridge (2014)
